# The influence of travel time on emergency obstetric care seeking behavior in the urban poor of Bangladesh: a GIS study

**DOI:** 10.1186/s12884-016-1032-7

**Published:** 2016-08-22

**Authors:** Rocco Panciera, Akib Khan, Syed Jafar Raza Rizvi, Shakil Ahmed, Tanvir Ahmed, Rubana Islam, Alayne M. Adam

**Affiliations:** 1 International Centre for Diarrhoeal Disease Research, Bangladesh (icddr,b), 68 Shahid Tajuddin Ahmed Sharani, Mohakhali, Dhaka 1212 Bangladesh; 2 James P. Grant School of Public Health, BRAC University, 5th Floor, (Level-6), icddr,b Building, 68 Shahid Tajuddin Ahmed Sharani, Mohakhali, Dhaka 1212 Bangladesh; 3 Institute of Development Studies (IDS), University of Sussex, Library Road, University of Sussex, Brighton, East Sussex BN1 9RE UK

**Keywords:** Emergency obstetric care, Health seeking, Urban, Travel time, GIS

## Abstract

**Background:**

Availability of Emergency Obstetric Care (EmOC) is crucial to avert maternal death due to life-threatening complications potentially arising during delivery. Research on the determinants of utilization of EmOC has neglected urban settings, where traffic congestion can pose a significant barrier to the access of EmOC facilities, particularly for the urban poor due to costly and limited transportation options. This study investigates the impact of travel time to EmOC facilities on the utilization of facility-based delivery services among mothers living in urban poor settlements in Sylhet, Bangladesh.

**Methods:**

A cross-sectional EmOC health-seeking behavior survey from 39 poor urban clusters was geo-spatially linked to a comprehensive geo-referenced dataset of EmOC facility locations. Geo-spatial techniques and logistic regression were then applied to quantify the impact of travel time on place of delivery (EmOC facility or home), while controlling for confounding socio-cultural and economic factors.

**Results:**

Increasing travel time to the nearest EmOC facility is found to act as a strong deterrent to seeking care for the urban poor in Sylhet. Logistic regression results indicate that a 5-min increase in travel time to the nearest EmOC facility is associated with a 30 % decrease (0.655 odds ratio, 95 % CI: 0.529–0.811) in the likelihood of delivery at an EmOC facility rather than at home. Moreover, the impact of travel time varies substantially between public, NGO and private facilities. A 5-min increase in travel time from a private EmOC facility is associated with a 32.9 % decrease in the likelihood of delivering at a private facility, while for public and Non-Government Organizations (NGO) EmOC facilities, the impact is lower (28.2 and 28.6 % decrease respectively). Other strong determinants of delivery at an EmOC facility are the use of antenatal care and mother’s formal education, while Muslim mothers are found to be more likely to deliver at home.

**Conclusions:**

Geospatial evidence points to the need to strengthen referral and emergency transport systems in order to reduce urban travel time, and establish or relocate EmOC facilities closer to where the poor reside. However, female education and antenatal care coverage remain the most important determinants of facility delivery.

## Background

Everyday around 800 mothers die worldwide due to complications during pregnancy and childbirth such as hemorrhage, infections and hypertensive disorders [1]. Most of these occur in low and middle-income countries and could be averted with appropriate precautionary measures [1]. The most recent data in Bangladesh indicate a Maternal Mortality Ratio (MMR) of 194 deaths per 100,000 live births as of 2010 [2], making Bangladesh one of only nine countries on track to achieve the primary target of Millennium Development Goal (MDG) for maternal health [3]. However, a further 63 % reduction in MMR is needed by 2030 to achieve Sustainable Development Goal number 3.1 of less than 70 deaths per 100,000 live births, set by the UN as part of the post-MDG global development framework.

Access to skilled attendance at delivery is advocated as the “single most important factor in preventing maternal deaths” due to life-threatening complications arising during delivery, amongst other factors such as efficient referral and promotion of antenatal care [4]. In Bangladesh, skilled attendance is synonymous with facility delivery, as only 3–11 % of home deliveries are attended by medically trained providers [3, 5]. Since the late 1980’s, a series of initiatives aimed at improving the quality and utilization of EmOC services have been implemented by the Government [6–8]. These efforts, together with the widespread use of antibiotics, the reduced travel time to facilities due to improved transport infrastructure and the increased availability of services, among others, have contributed to a substantial decrease in MMR from 322 per 100,000 live births in 1998 to 194 per 100,000 live births in 2010 [3]. Despite this progress, EmOC facilities continue to be underutilized due to preferences for home delivery, often with unqualified practitioners and delays in decision-making to seek care, time taken to reach the facility and waiting time at the facility (the “three delays”) [9–14]. Consequently, the proportion of women delivering at an EmOC facility in Bangladesh remains low (29 %), and even lower (10 %) amongst the poorest socio-economic portion of the population [3].

Although it is well-established that urban women have a higher odds of delivering at a facility than rural women [5, 15], inequities in availability, accessibility and utilization lie behind urban averages [16]. Unlike rural areas in Bangladesh, where a formal public health system under the Ministry of Health and Family Welfare enables the delivery of primary care with clear referral linkages to secondary and tertiary services, these do not extend to urban areas where, under the jurisdiction of the Ministry of Local Government, urban primary healthcare systems are in relative disarray [16]. Over 70 % of those living in poor urban settlements do not have access to a governmental health facility within a 2 km distance and rely mostly on NGO and private providers [16]. Lack of coordination, inadequate planning and the unregulated growth of the private sector has contributed to services of uncertain quality and inequitable coverage, resulting in a significant gap in the utilization of EmOC between women residing in slums (37 %) and in non-slum urban areas (65 %) [16]. Reflecting these inequities, the burden of maternal mortality is heaviest among the poor, especially those residing in poor urban settlements [2, 17].

Variations in the degree to which the urban poor can access and utilize EmOC may arise for reasons of geographic distance to facilities with delivery capacity, as well as socio-cultural and economic differences [11, 16–18]. To date, the majority of studies investigating the determinants of EmOC utilization have concentrated on household, socio-cultural, demographic and individual characteristics of mothers. Variables of perceived benefit/need and physical accessibility, such as distance to EmOC facilities, have received relatively less attention [11, 19]. Existing evidence suggests that distance from EmOC facilities may affect the “first delay” indirectly, by creating a disincentive to seek care, and the “second delay” directly, by increasing the time before services are actually received [10, 11, 13, 20]. In Bangladesh, the most recent data report that the first and second delays represent a cumulated median of 6.6 h for those living in poor urban settlements, severely hindering timely access to effective EmOC, especially in life-threatening situations [17]. Although urban maternal and child health programs like BRAC’s *Manoshi* project have been very effective in reducing the first delay (i.e., the decision to seek care), reductions in the second delay (i.e., the time taken to reach facilities) have been more difficult to achieve [21].

Evidence on the extent to which distance acts as a deterrent to facility delivery in Low and Middle Income Countries (LMIC) is mixed. A number of studies indicate a negative impact [10, 11, 13, 22–28], especially when labor starts unexpectedly at night and in the absence of transport options. In other studies no effect of distance was observed [29, 30], or interesting inverse effects were reported [31–33], such as women from a remote village in India choosing to deliver at a distant private hospital because “the distance from their village to the primary health center made them skeptical about delivering at home in case complications occurred” [31]. The deterrent effect of distance has been observed to be stronger when combined with lack of transport and poor roads [34, 35], and low perceived quality of care [35, 36]. Clearly, understanding the effects of distance on facility utilization is complex, given its implicit associations with real and perceived deterrents including poverty, inadequate road infrastructure, weak communication systems, perceived quality of care, limited access to information and adherence to traditional values that are difficult to measure quantitatively [37].

According to several recent systematic reviews [11, 19, 38], most of the work that explored the impact of distance on utilization of EmOC in LMIC has been qualitative in nature. Comparatively few studies have been based on actual measurements of geographic distance or travel time [11], and these are nearly exclusively focused on rural areas. GIS-based studies in rural areas of Ethiopia, Ghana, Burkina Faso, Mali, Malawi, Zambia, Bangladesh and Cambodia consistently found that increasing distance to facility acts as a significant deterrent to facility delivery [13, 23–28], and is also frequently associated with higher risk of neonatal and maternal mortality [13, 26, 39], even after adjustment for various socio-economic factors.

This literature review highlight the dearth of evidence on the impact of geographic barriers to EmOC utilization in urban settings, due in part to the lack of merged geo-referenced datasets on health-seeking behavior and health facility locations and characteristics, and the perception that the relatively small distances involved in urban transportation might not represent a significant obstacle to facility delivery [11, 19, 38]. However, as shown by a urban study in Brazil [40], extended travel time, due to poor road infrastructure and traffic congestion typical of developing megacities, can pose substantial difficulties in accessing EmOC despite short geographic distances, particularly when combined with poverty and lack of transport options.

In order to inform urban planning actions required to reduce maternal mortality rate, it is therefore crucial to understand and quantify the impact of distance on utilization of EmOC specifically in urban settings, and particularly among the urban poor who are the most affected by geographic barriers. The present study investigates the utilization of EmOC in an urban district of Bangladesh by drawing on methods from existing geographic studies [13, 23, 39, 41, 42], and by combining geo-referenced individual/household-level data capturing choice of delivery place with geospatial information on EmOC facilities locations and transportation networks. The present study therefore contributes to filling the existing knowledge gap around understanding the causes of low EmOC utilization in low income urban settings. Specifically, the research questions addressed by this study are:

1. To what degree does geographic distance between poor urban settlements and available EmOC facilities affect the utilization of facility delivery?
2. Is the impact of geographic distance on the utilization of facility delivery dependent on the type of facility (i.e., public, private or NGO-managed facilities)?
3. How does the impact of geographic distance on the utilization of facility delivery services compare with the influence of other known determinants of facility utilization in poor urban settlements?

**Fig. 1:**
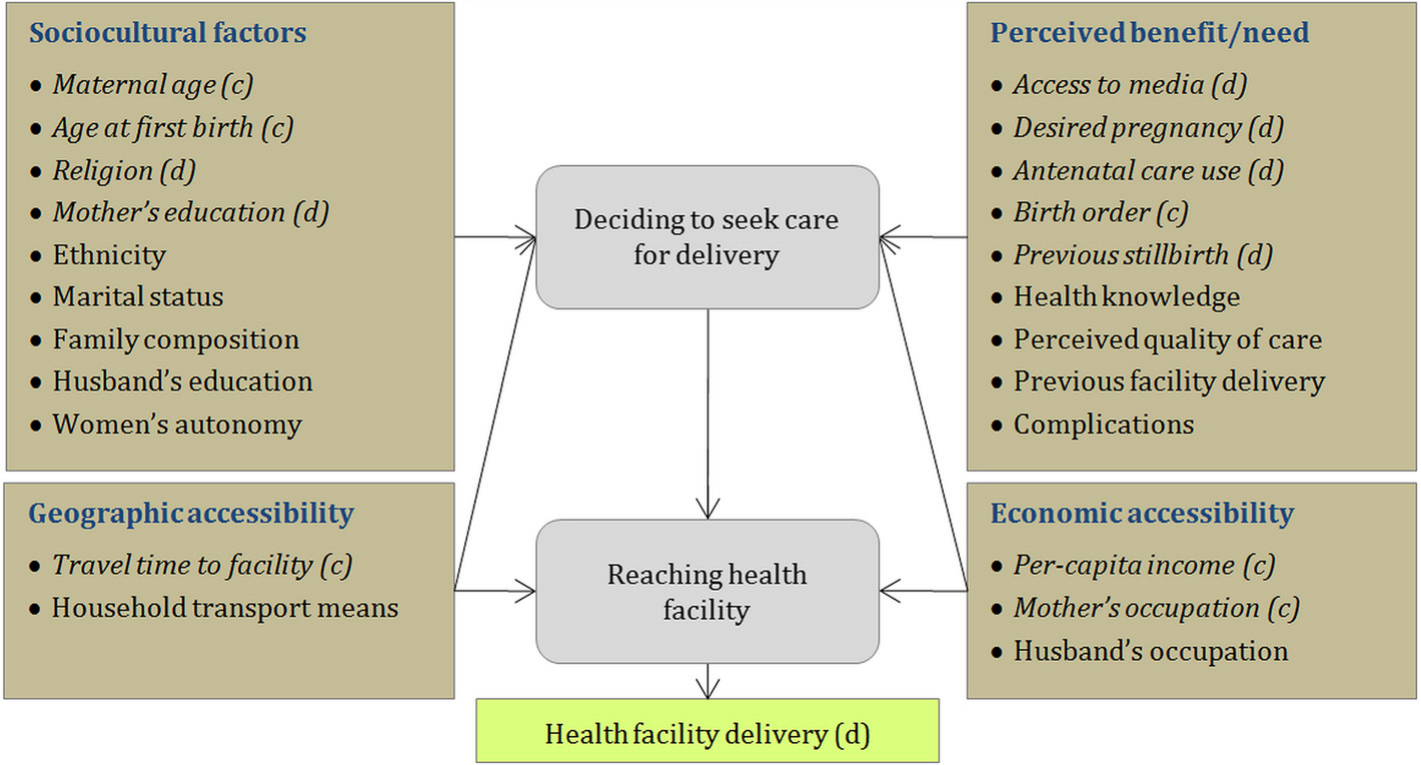
Conceptual framework of the determinants of facility delivery (Adapted from Gabrysch and Campbell, [11]). (label) Variables used in the analysis are indicated in italic font. Variable type is indicated as (c) for continuous and (d) for dichotomous variables

Geographic Information Systems (GIS) analysis is used to calculate travel times between poor urban settlements and facilities along road networks, and to assess the level of spatial association between choice of delivery place and facility location. Multivariate logistic regression(s) methods are employed to specify the impact of travel time by controlling for other relevant socio-cultural and economic determinants known to potentially affect health-seeking behavior. The conceptual framework guiding the analysis of the determinants of facility delivery is summarized in Fig. 1.

## Methods

The data used in this study derives from a healthcare seeking behavior survey conducted among the urban poor in Sylhet City Corporation (SCC), Bangladesh, in 2013, together with a comprehensive geo-referenced census of health facilities conducted in the same year. SCC is the capital of Sylhet district in northeastern Bangladesh, with an area of 26.5 km^2^ and a population of over 500,000 people distributed across 27 wards and 210 mahallas. Sylhet Division has the lowest rate of EmOC facility utilization (21 %) in Bangladesh, and sharp wealth inequities, ranking second nationwide for both the lowest and highest wealth quintiles [5].

### Healthcare seeking behavior data

The Healthcare Seeking Behavior (HSB) survey was specifically designed to identify the determinants of health care seeking, including demographic and socio-economic factors as well as geographic factors such as distance to facilities and transportation options. The HSB contained information on 610 deliveries of urban poor women from 39 different clusters in SCC that occurred within one year preceding the survey. The initial sample frame was identified using data from the BRAC *Manoshi* program, which operates maternal and child health services through three branch offices in SCC, each serving a specific geographic zone. A two-stage cluster sampling approach was utilized. A cluster was defined as a group of 200 households served by a community health worker each from BRAC *Manoshi;* there were 189 such clusters identified. The first stage involved randomly selecting 39 clusters from each geographic zone and, within each cluster, undertaking a comprehensive household listing. The second stage involved a random selection of married women who delivered in the past year as identified from the household listing. Sample-size was calculated based on findings from the Bangladesh Urban Health Survey (2006), indicating that 48 % of slum dwelling women of reproductive age sought care for maternal complications. A design effect of 1.5 and a 10 % non-response rate were assumed, leading to a sample size of 633 women. Information on household socio-cultural factors, community mobilization, social capital and individual health seeking behavior were collected using a structured questionnaire. Geospatial location data for the household clusters were also collected as a part of the survey. Individual household data were weighted to adjust for differences in the probability of selection and interview between cases, in order to get a better representation of the target population.

### Health facilities survey data

A GIS-based census of public, private and NGO health facilities in SCC was conducted from November 2012 to February 2013. In addition, data were collected on type of facility, management entity, facility focus, service hours, staffing pattern, qualifications and training, services offered and basic infrastructure. The facilities that provided delivery care were classified into two categories, Basic and Comprehensive EmOC, based on signal functions defined by the WHO [43]. The signal functions used to identify Basic EmOC facilities are: administer parenteral antibiotics, administer uterotonic drugs (i.e. parenteral oxytocin), administer parenteral anticonvulsants for pre-eclampsia and eclampsia (i.e. magnesium sulfate), manually remove the placenta, remove retained products (e.g. manual vacuum extraction, dilation and curettage), perform assisted vaginal delivery (e.g. vacuum extraction, forceps delivery) and perform basic neonatal resuscitation (e.g. with bag and mask).

In addition to these signal functions, if a health facility had provisions for cesarean section and blood transfusion, it was classified as a Comprehensive EmOC facility [43]. As assisted vaginal delivery, using either forceps or vacuum extractor, is the least available form of delivery in Bangladesh [44], we omitted this signal function in classifying the EmOC facilities. Upon scrutiny, a total of 19 Comprehensive EmOC facilities were identified. These included 14 privately managed hospitals, 3 NGO clinics and 2 public hospitals. Basic EmOC facilities were mainly comprised of BRAC delivery centers, established under the *Manoshi* program and intentionally located in close proximity to poor urban settlements (<1 km). Such proximity makes the analysis of the effect of distance on EmOC utilization not meaningful for BRAC centers. Therefore this study focuses on utilization of Comprehensive EmOC facilities only (public, private or NGO), thereby reducing the sample size to 514 women. This exclusion does not significantly affect the relevance of the findings since the utilization of BRAC centers was relatively low among the sampled women (11 %).

The HSB survey was approved by the Institutional Review Board of the International Centre for Diarrhoeal Disease Research, Bangladesh (icddr,b). For health facility survey, special permission was sought from the Director General of Health Services, Bangladesh. All interviews were preceded by detailed explanation of the research objectives and methods, participant rights and confidentiality concerns. Written informed consent was sought for all interviews; in case of healthcare seeking behavior survey, parents/guardians of children under 15 years were interviewed.

### Conceptual framework and variable classification

Based on an extensive review of the determinants of delivery service choice, the conceptual framework of Gabrysch and Campbell [11] was used to help identify relevant and evidence-based explanatory variables Fig. 1. As indicated in the Figure, only those explanatory variables appearing in italic font are available in the HSB dataset and are included in analysis. Mother’s occupation was treated as a dichotomous variable based on her participation (1) or not (0) in wage employment in the 4 weeks previous to the HSB survey. Similarly, Mother’s education was determined based on whether she had received any (1) or no (0) formal education (school/madrasa), as data on ‘years of schooling’ presented a significant percentage of missing values. A value of 1 for religion indicates Muslim, while 0 includes all other religions. For access to media, a value of 1 was assigned for daily, weekly or at least infrequent access to at least one media channel (either newspaper, radio or television). Antenatal care use, also modeled as a dichotomous variable, was ascribed a value of 1 if the mother received at least one check-up during pregnancy (and 0 otherwise). This choice was justified by the fact that only a limited number of mothers (19.3 %) had received the number of antenatal consultations recommended by WHO (> = 4). Although information on ethnicity was also collected as part of the HSB survey, the limited number of ethnic minorities in the study area (1.2 %) does not warrant inclusion of the variable in the analysis.

### Travel time calculation

Distance between each cluster and the closest EmOC facility was calculated as the travel time along the shortest route using ArcGIS and detailed road network data. In line with the common practice in GIS studies of geographic accessibility [45–47], travel time was estimated using travel speed estimates for each road network section, differentiated using available information on the road section type (primary arteries, sub-arteries and secondary roads). Realistic estimates of travel speeds for each road type were obtained using traffic data from an independent traffic survey, which reported total travel times between 5 major intersections in SCC [48]. Using these data, travel speeds for different road types were estimated using an optimization approach that minimized the difference between travel times between major intersections and those calculated using ArcGIS Network Analyst. The resulting travel speed estimates (11.5 km/h for arteries and sub-arteries, and 5 km/h for secondary roads) were then assumed constant throughout the road network. This approach to travel speed estimation allowed us to account for more realistic travel times than what would be possible using car speed limits, which is the usual practice in GIS studies of geographic accessibility to health care in developed countries [45–47]. This approach was deemed more applicable to dense urban areas of Bangladesh where highly congested traffic conditions predominate, and where gas-powered 3-wheelers and rickshaws are the more common mode of transportation among poorer urban residents.

For each cluster, travel times to the nearest EmOC facility of each type (NGO, private or public) and the travel time to the EmOC facility nearest to the cluster (regardless of the type of facility) were saved into four travel time vectors. Each household was then assigned the travel time of the relevant cluster. Given that the HSB questionnaire only sought information on the type of facility where EmOC was delivered (i.e., either NGO, public or private), but not the actual facility name, an assumption was made that the travel time affecting the choice of delivery place was that between the cluster and the type of facility utilized that was nearest to the cluster. As previously mentioned, this may not be true as distant facilities may be chosen over nearer ones due to differences in perceived quality of care [11]. The validity of this assumption will be further discussed in later sections.

### Spatial and statistical analysis

The spatial characteristics of the study area and the degree of spatial correlation between travel time and choice of delivery place were quantified using Global Moran’s I (section 3.1). This index measures the spatial autocorrelation between the % choice of facility delivery for each cluster and the respective travel time to nearest EmOC along a scale between −1 (inverse correlation) and +1 (positive correlation). Since spatial autocorrelation cannot be calculated for dichotomous variables, individual mother’s choices were aggregated and expressed as % of facility delivery over total deliveries for each cluster before calculating the index. A fixed 4-neighbour criteria was used for the index calculation in order to account for the variable spatial density among the sampling clusters.

To investigate differences between mothers delivering at home and those delivering at EmOC facilities, various explanatory variables (i.e. socio-cultural and economic characteristics, travel time) were explored using bivariate analysis (see section 3.2). Multivariate binomial and multinomial logistic regression models were then applied to assess the independent impact of travel time to the nearest EmOC facility on choice of delivery place (section 3.3). This analysis was conducted in two steps. First, the dichotomous outcome of interest was specified as the choice of delivery at a facility of any type (public, private or NGO) versus delivery at home. In this instance, a multivariate binomial logistic regression model was used, with the primary explanatory variable being the travel time to the closest EmOC facility of any type, and the other socio-economic and cultural factors included as covariates.^1^ As a second step, a multinomial regression model was estimated where the dependent variable had four outcomes/alternatives (delivery at home, or at public, private and NGO facility). Using McFadden’s choice model, travel time was included as an alternative-varying regressor, with other covariates acting as alternative-invariant regressors. Thus specified, this model allowed us to investigate whether the impact of travel time (and other covariates^2^) on delivery service utilization depends on the type of facility. Both multivariate binomial and multinomial logistic regression models also controlled for the effect of the confounding factors discussed earlier. In addition, as a robustness check for the potential correlation across mothers within a given cluster, we estimated the clustered standard errors of model coefficients, and assess how this affected their significance levels. This is a common practice when analyzing data drawn from clustered samples [49–51].

Throughout the analysis, key variables are expressed as z-scores (or ‘standardized’ scores) to facilitate interpretation. Z-scores express the variation of a variable with respect to the mean of the variable’s population, in units of standard deviations of the population. Their use permits the interpretation of regression models as a change of odds in units that reflect the variable’s standard variability in the sample.

## Results

### Spatial analysis

**Fig. 2:**
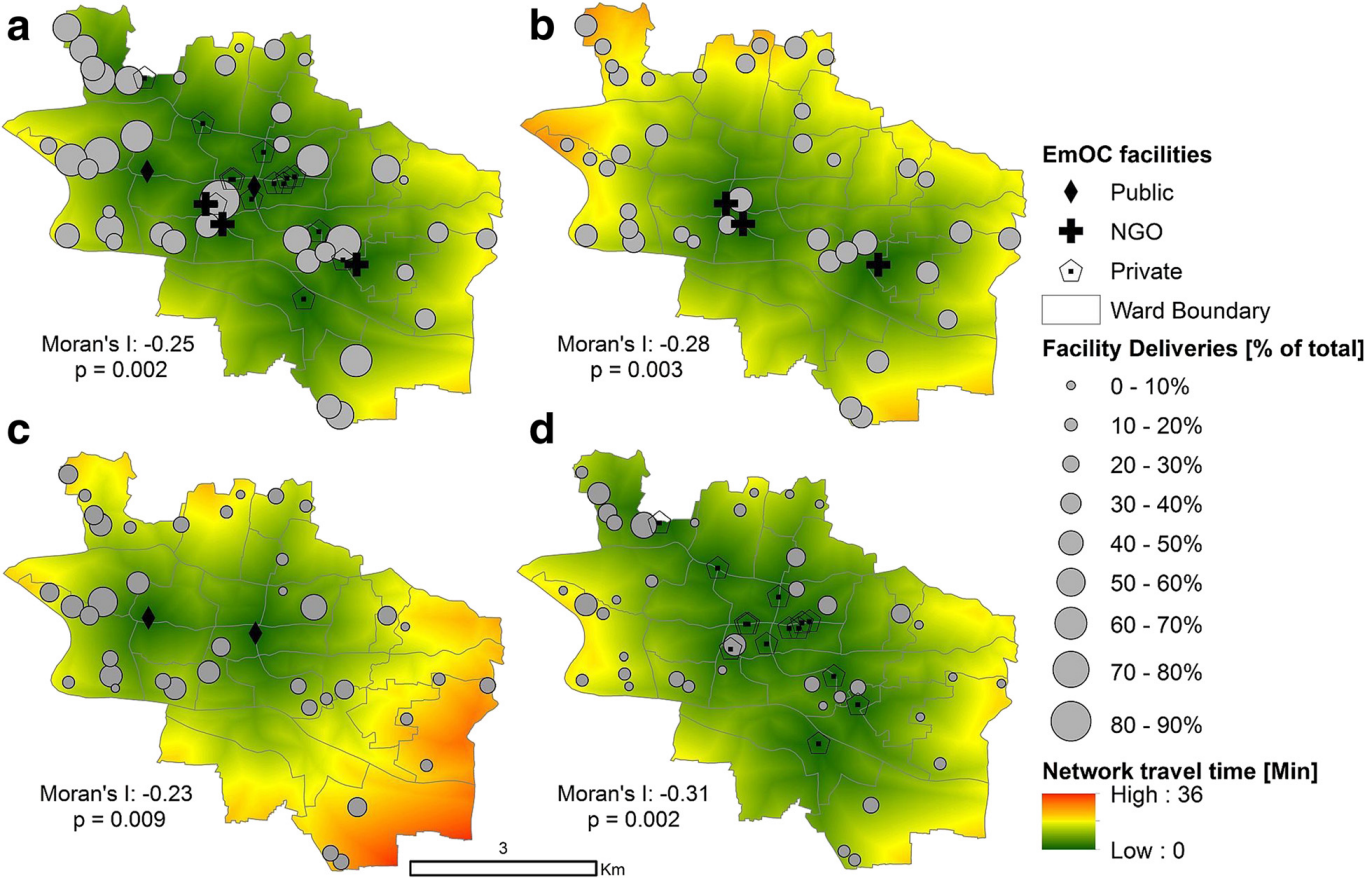
Spatial distribution of EmOC facilities and sampling clusters in Sylhet City Corporation. (label) Different panels show all facility types (panel **a**) and individually NGO (**b**), public (**c**) and private facilities (**d**). The size of the clusters reflects the ratio of facility deliveries to total deliveries for each cluster (in %). The shortest travel time calculated along the road network is shown as background. Statistics for the Global Bivariate Moran’s I index shown are calculated between the percentage of facility deliveries aggregated at cluster level and the network travel time calculated at the cluster center

Figure 2 displays the spatial distribution of EmOC facilities and sampling clusters in SCC, together with a map displaying the distribution of travel time to the nearest EmOC facility. EmOC facilities are visibly concentrated towards the city center, while sampling clusters are more dispersed across SCC. Travel times are in the 0–30 min range for the entire SCC. Although the distribution of facility deliveries (%) does not show an obvious spatial pattern when all facility types are included (Fig. 2a), when specific facility types are considered, some interesting patterns emerge relative to the travel time maps. Deliveries at NGO facilities are substantially lower in the western-most clusters, in association with larger travel times (Fig. 2b), and higher at public facilities, consistent with lower travel times (Fig. 2c). The eastern-most clusters of SCC are mainly served by nearby NGO facilities. A more marked association between the percentage of deliveries occurring at private facilities and the spatial distribution of such facilities is apparent relative to NGO or public facilities, with higher deliveries concentrated in the city center and the furthest ward to the north-west.

Figure 2 presents the spatial correlation between the percentage of facility deliveries and the travel time using the Global Moran’s I spatial correlation index. All Moran’s indices in Fig. 2 are negative and statistically significant (*p* < 0.01), indicating a significant (inverse) spatial association between facility delivery and travel time. In particular, results demonstrate that a statistically significant spatial association exists between locations with high travel time and low facility delivery (and vice versa). The highest correlation is observed for delivery at private facilities (Moran’s I -0.31), and the lowest for public facilities (Moran’s I -0.23). When Moran’s I coefficients are expressed in terms of travel time, a 5-min increase in travel time is associated with a 5.5 % decrease in the percentage of deliveries at any facility (over total cluster deliveries), and a 2.5, 2.8 and 3.7 % decrease in deliveries at public, NGO and private facilities respectively. It should be noted, however, that these figures are aggregated at the cluster level (as opposed to the individual household level), and represent a measure of global association between travel time and % facility delivery across the study area.

**Fig. 3:**
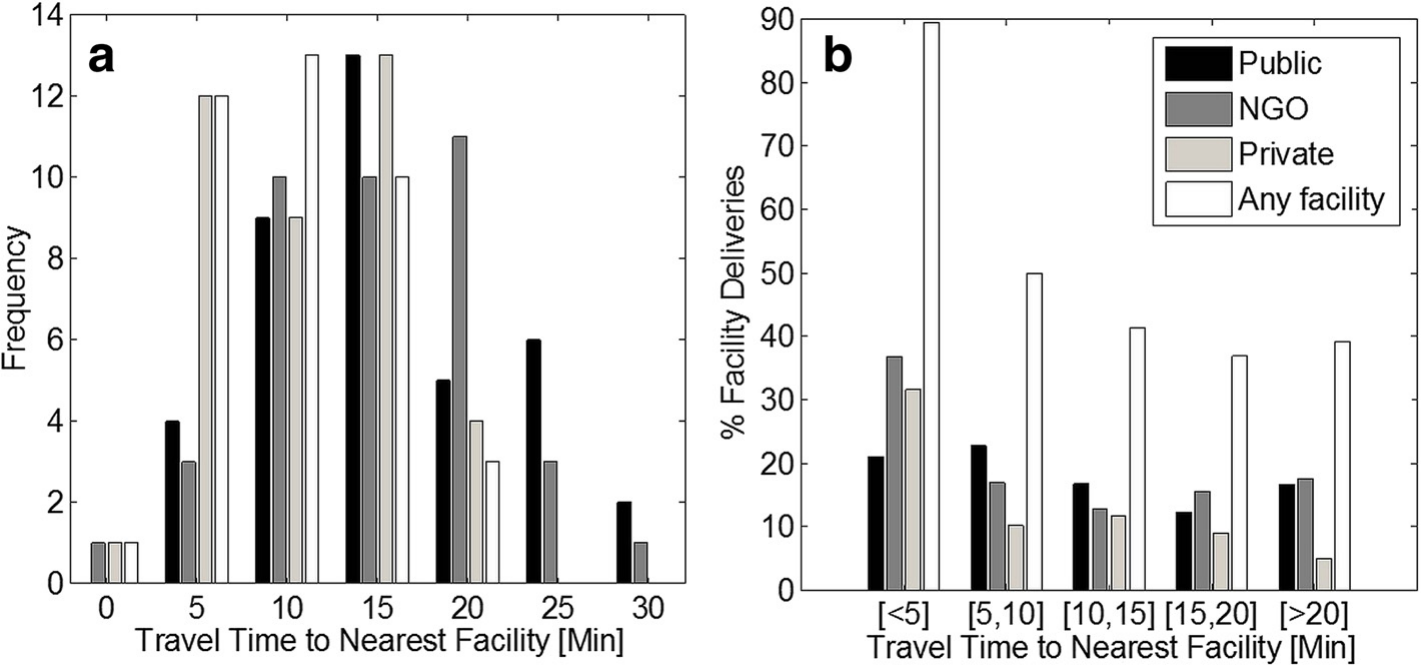
Effect of travel time on utilization of EmOC facilities. (label) (**a**) Histograms of network travel times between the 39 sampling clusters and 19 EmOC facilities and; (**b**) Variation of % facility delivery by travel time brackets, shown for different facility types

Despite the statistical significance of the Moran’s I coefficient, the relatively low values of the index (which can reach +/-1 for highly correlated variables) indicate the potential presence of confounding factors in the relationship between travel time and choice of delivery. This is particularly apparent for deliveries at NGO and public facilities. To further highlight travel time dependence, Fig. 3a displays a histogram depicting the distribution of travel times between sampling clusters and EmOC facilities. It is important to note that travel times to private facilities are shorter and skewed towards lower values, ranging from 0 to 20 min. This is a reflection of the large number of private EmOC facilities in SCC. Conversely, travel times to the (fewer) public and NGO facilities are more normally distributed and exhibit a wider range (0–30 min). In Fig. 3b, the relationship between choice of delivery place and travel time is explored by plotting the percentage of deliveries at a facility for increasing intervals of travel time (to the nearest EmOC facility). The plot, which distinguishes between delivery at public, private, NGO or any facility, suggests a substantial decline in delivery with increasing travel time when no distinction is made between facility types. For the shortest travel time interval “[<5 min]”, nearly 90 % of deliveries are carried out at facilities. With increasing travel times, deliveries at facilities drop to 40 %. This trend is reflected across all facility types, but appears to be more prominent for delivery at private and NGO facilities, consistently with the spatial analysis discussed earlier.

The analysis presented in this preliminary section indicates a significant impact of travel time on choice of facility delivery that warrants further investigation. In the next section the confounding factors indicated in Fig. 1 are introduced in a more sophisticated inferential analysis.

### Descriptive statistics

Table 1 explores whether socio-cultural or economic differences exist between mothers delivering at home or at public, private or NGO facilities using ANalysis Of VAriance (ANOVA). More than half of the mothers interviewed delivered at home (56.5 %). Of the 43 % of mothers reporting facility delivery, the majority used public (17 % of total deliveries) and NGO facilities (16 %), with 10 % opting for private facilities. Table 1 confirms that mothers delivering at home belonged to clusters that were, on average, more distant (i.e., longer travel time) from any type of facility. The difference in mean travel time between these two groups was minimal for public facilities (0.5 min) and up to 3.3 min for private facilities. Although it might seem small, a 3.3 min difference corresponds to a distance of 0.6 km at the travel speed permitted in typical SCC traffic conditions [48]. The first three rows of Table 1 also indicate that mothers do not necessarily deliver at the facility closest to their cluster. For example, mothers delivering at public facilities were on average closer (10.8 min) to private facilities than they were to public facilities (14.9 min), and the same holds true for mothers opting for a NGO facility delivery. This is not surprising since these facilities are not considered perfect substitutes of each other due to differences in perceived quality and cost.

**Table 1:** Socio-cultural and economic characteristics of women by form of delivery in the last 6 months

Variables [units]	All deliveries^a^	Home deliveries^a^	Facility deliveries^a^			
			Any type	Public	NGO	Private
Travel time [min]						
To nearest public facility	15.6	16.4	15.3**	14.9**	16.5	14.0*
To nearest NGO facility	15.0	15.6	14.6***	14.5	13.7**	14.5
To nearest private facility	11.0	11.6	10.2***	10.8	10.7	8.3***
Sociocultural factors						
Age [years]	24.9 (15–45)^b^	24.9	24.8	25.4	24.1	25.4
Age at first birth [years]	19.4 (12–35)^b^	18.7	20.3***	20.7***	19.3	21.4***
Religion [%]	83.7	90.5	73.2***	74.2***	75.9***	67.3***
Mother’s education [%]	68.3	57.0	82.9***	79.6***	80.5***	92.7***
Perceived benefit/need						
Access to media [%]	85.0	81.9	88.9**	87.1	87.4	94.5**
Pregnancy wanted [%]	63.0	61.6	64.4	71.0	51.7*	74.5*
Antenatal care use [%]	82.2	72.0	95.0***	96.8***	90.8***	100.0***
Birth order [#]	2.3 (0–8)^b^	2.5	1.86***	1.8***	2.1	1.6***
Previous stillbirth [%]	9.5	8.8	11.0	8.6	11.5	12.7
Economic accessibility						
Per-capita income [1000BDT]	2.3 (0.1–17)^b^	1.9	2.7***	2.9***	2.3**	3.0***
[US$]	29.7 (0.8–215)^b^	24.5	34.8***	37.4***	29.6***	38.7***
Mother’s occupation [%]	12.3	11.0	14.0	18.5*	10.3	12.7
Delivery Service Use		56.5	43.5	17.2	16.1	10.2
Sample size: 514 mothers						

Bold text indicate values statistically significant to better than *p* < 0.1

* *p* < 0.1; ** *p* < 0.05; *** *p* < 0.01, ANalysis Of VAriance (ANOVA) *p*-values indicating whether the mean values for each delivery group are statistically different (higher or lower) relatively to the home delivery group

^a^Average values of each respective variable in each delivery group

^b^For numerical variables, the range of values in the sample is also indicated

Reference categories: Mother’s occupation - participated in wage employment in the 4 weeks previous to survey (1), 0 otherwise; Mother’s education - received any (1) or no (0) formal education in school or madrasa; Religion - Muslim (1), all other religions (0); Access to media - daily, weekly or infrequent access to at least one media channel (either newspaper, radio or television) (1), 0 otherwise; Antenatal care use - received at least one check-up during pregnancy (1), 0 otherwise; Previous still birth and pregnancy wanted - positive (1) or negative (0) answers

Table 1 also provides some interesting insights about the characteristics of mothers choosing to deliver at different facility types. Per-capita income, formal education and use of antenatal care were significantly greater for mothers delivering at all facility types relative to mothers delivering at home: income was 800 BDT higher (approximately ~10 US$, a considerable difference given the 29 US$ mean per-capita income of the sample household), and rates of formal education and use of antenatal care were 26 and 23 percentage points greater respectively. The proportion of mothers from Muslim households delivering in facilities was also significantly lower than those in non-Muslim households (by 17 percentage points). These differences are even more marked for women using private facilities compared to public and NGO services. Mothers delivering at private facilities had higher (mean) income (by nearly 1100BDT, or 14 US$ per-capita), were more educated (by 35 percentage points) and were less likely to be Muslim (by 23 percentage points) than mothers choosing to deliver at home. Moreover, the percentage of women accessing antenatal care was highest among users of private facilities, compared to other facility types. Similarly, desired pregnancy and access to media were significantly higher among the private facility group compared to mothers delivering at home (both by 13 percentage points).

In contrast, mothers delivering at NGO facilities were characterized by significantly lower reports of desired pregnancy than those delivering at home (by 10 percentage points), or at public and private facilities. The greatest proportion of Muslim mothers delivered at NGO facilities (76 %). Finally, mothers delivering at public facilities were employed to a significantly greater extent than mothers choosing home, NGO or private facilities. Mother’s age and stillbirth did not differ significantly between mothers delivering at facilities or at home.

Results from statistical analysis points to an influence of travel time on place of delivery in a manner consistent with the spatial correlations discussed earlier. Most importantly, they highlight the significance of other factors in the choice of delivery place, and the differing impacts of increasing travel time and confounding factors depending on the facility type. Logistic and multinomial regression analysis were therefore conducted to isolate the impact of travel time on choice of delivery and control for other socio-economic and cultural confounding factors.

### Logistic regression

Table 2 reports the odds ratios from the binomial logistic regression models for the case of delivery at a facility of any type (or at home). Results demonstrate a significant inverse relationship between travel time to an EmOC facility and the probability of delivery at a facility (at the 5 % significance level). Given that travel times are introduced in the regression model as normalized z-scores, these results translate into one standard deviation increase in the travel time to the closest EmOC facility (approximately 5 min) being associated with a decrease in relative probability of delivering at a facility of at least 21.4 % (0.786 odds ratio, 95 % CI: 0.628–0.984) when other factors are controlled for (see column (2)). In other words, greater travel time is associated with a higher likelihood of a delivery taking place at home in lieu of a formal health facility. It should be highlighted that, although the 5-min figure expresses the result of the logistic regression in terms of the ‘natural’ variability of the travel time sample, it captures the impact of travel time over the full observed range of travel time (approximately 0–30 min, see Fig. 3). Use of antenatal care, mother’s education and religion were also found to be significant predictors of where delivery occurs. Mothers who sought antenatal care and possessed some formal education were respectively (almost) 6 and 4 times more likely to deliver at a facility rather than at home. On the other hand, the odds of a facility delivery were almost 54 % lower for Muslim mothers, and those who had their first delivery at a later age had a slightly higher (14 %) relative probability of facility delivery. While some regression results are consistent with prior statistical analysis, estimates differ in terms of the effect of stillbirth, which almost doubles the likelihood of facility delivery. Moreover, per-capita income, birth order and access to media had no significant effect on where delivery occurs. Mother’s paid employment had a positive impact on utilization of facilities, although this was not found to be statistically significant.

**Table 2:** Binomial logistic regression of factors predicting delivery at EmOC facility

	Delivery at EmOC facility of any type	
	Odds ratio (95 % confidence intervals)^c^	
Variables	Model^a^	Model^b^
Travel time [z-score]	0.756*** (0.625–0.914)	0.786** (0.628–0.984)
Age at first birth		1.139*** (1.066–1.216)
Religion		0.461** (0.252–0.844)
Mother’s education		3.764*** (2.090–6.779)
Access to media		1.233 (0.600–2.533)
Desired pregnancy		0.769 (0.466–1.271)
Antenatal care use		5.736*** (2.300–14.304)
Birth order		0.928 (0.743–1.159)
Previous stillbirth		1.870* (0.925–3.782)
Per-capita income		1.110 (0.971–1.268)
Mother’s occupation		1.685 (0.862–3.296)
Constant	0.853 (0.704–1.034)	0.008*** (0.001–0.064)
Observations	540	514
Pseudo R-squared	0.0131	0.212

Model specifications

* *p* < 0.1; ** *p* < 0.05; *** *p* < 0.01. Bold text indicate values statistically significant at the 1 % level

^a^Only travel time to the nearest EmOC facility as explanatory variable

^b^Confounding factors as additional explanatory variables

^c^Use of clustered standard errors did not affect the significance levels of the estimated coefficients

Tables 3 and 4 report results from multinomial regression models that examine the determinants of delivery at different types of facility. Consistent with prior statistical analysis, there is a significant inverse relationship (at the 1 % level) between travel time and probability of facility delivery, irrespective of type of facility. When estimates are adjusted to reflect the change in odds in response to an increase in travel time of 5 min, a 30 % decrease (0.655 odds ratio, 95 % CI: 0.529–0.811) in the relative likelihood of delivery at any facility is apparent. When different types of facilities are considered, a 5-min increase in travel time has the greatest negative effect on the utilization of private facilities (as opposed to home), decreasing the livelihood of delivery by 32.9 %, compared to public (28.2 %) and NGO (28.6 %) facilities respectively. This supports earlier findings from spatial correlation and statistical analysis. While the results of spatial correlation in section 3.1 cannot be compared in absolute terms with the figures presented here, since they are aggregated at the cluster level and represent a % delivery ratio rather than a change in probability, they nevertheless strongly support the stronger association observed between travel time and delivery at private facilities compared to NGO or public facilities.

**Table 3:** Differential (marginal) impact of travel time from multinomial logistic regression

% Change in relative probability of delivery with a 5-min increase in travel time^a^	
Delivery at any facility	−30.0
Public facility delivery	−28.2
Private facility delivery	−32.9
NGO facility delivery	−28.6

^a^Estimates derive from a statistically significant (at the 1 % level) odds ratio of travel time in the multinomial model, namely 0.655 (95 % CI: 0.529–0.811)

**Table 4:** Multinomial logistic regression of delivery at a public, private or NGO facility (McFadden’s choice model)

	Type of delivery facility		
	Public	Private	NGO
Variables	Adjusted odds ratio (95 % Confident Interval)^a^		
Age at first birth	1.185*** (1.085–1.295)	1.240*** (1.122–1.370)	1.056 (0.953–1.170)
Religion	0.362*** (0.173–0.755)	0.271*** (0.120–0.612)	0.442** (0.214–0.913)
Mother’s education	2.565*** (1.256–5.239)	6.047*** (1.844–19.832)	5.565*** (2.286–13.546)
Access to media	1.275 (0.508–3.197)	7.052** (1.558–31.913)	0.875 (0.351–2.182)
Desired pregnancy	1.163 (0.576–2.348)	0.706 (0.321–1.549)	0.516* (0.263–1.013)
Birth order	0.924 (0.673–1.267)	0.716* (0.496–1.033)	0.969 (0.695–1.350)
Previous stillbirth	1.204 (0.465–3.113)	2.685* (0.959–7.524)	1.837 (0.684–4.936)
Per-capita income	1.160* (0.993–1.356)	1.114 (0.917–1.353)	1.060 (0.900–1.248)
Mother’s occupation	2.197** (1.018–4.744)	1.694 (0.554–5.182)	1.028 (0.440–2.400)
Observations	514		
Log Likelihood	−4594		

* *p* < 0.1; ** *p* < 0.05; *** *p* < 0.01. Bold text indicate values statistically significant to *p* < 0.1

^a^Use of clustered standard errors did not affect the significance levels of the estimated coefficients

Consistent with results from binomial regression, mother’s formal education is a significant predictor of place of delivery, particularly for private facilities where it increased the odds six fold (Table 4). Muslim mothers were much less likely to deliver at a facility regardless of its type, but more so for private facilities, in which case being Muslim resulted in 73 % lesser likelihood of delivering at a facility as opposed to home. While engagement in wage employment seemed to matter only for the odds of delivery at a public facility, access to media was associated with a sevenfold increase in the odds only when it concerned private health facilities. Experience of stillbirth and birth order were influential (albeit in different directions; a 2.7-fold increase for the former and a 30 % decline for the latter), but solely for the case of delivery at private facilities. Conversely, unwanted pregnancies were twice as likely to be associated with delivery at NGOs, but had no significant bearing on the odds of delivery at public and private facilities. Somewhat surprisingly, income was not found to be a significant factor in determining the place of delivery in any of the multinomial models. It should be noted, however, that sample households were all located in settlements specifically identified as poor, and therefore the variance in income is lower than that expected for the general urban population.

## Discussion

Findings from the health-seeking behavior survey on which this analysis is based suggested that only 40 % of poor urban women having recently given birth planned to deliver at a facility (Islam R, Rizvi SJR, Ahmed R, Hillgrove T, Health care seeking in poor urban settlements in Sylhet City Corporation- a quantitative survey. 2014. icddr,b & GIZ. Unpublished report). This figure lies between the national average for urban (60 %) and rural areas (23 %) [5]. Those who planned for a facility delivery preferred public (17 %) and NGO facilities (16 %) over private (10 %), and valued proximity (39 %), followed by perceived friendliness of the health provider (35 %) and low cost (34 %) as the most important criteria informing their choice. The most common mode of transport used to get to the facility was natural gas-powered 3-wheelers (54 %), followed by rickshaw (32 %), foot (9 %), and ambulance (2 %).

Linking geo-referenced health seeking data with geospatial information on health facilities and transportation networks, this paper explored the influence of travel time to the nearest Emergency Obstetric Care (EmOC) facility on the likelihood of facility delivery in poor urban settings. Despite the density of service provision in urban areas, findings indicate that the geographic location of EmOC services (in terms of network travel time) has a significant impact on the utilization of facility delivery by the urban poor. Most strikingly, when all other known determinants of use of EmOC facilities are controlled for, a one standard deviation increase in the travel time to the closest EmOC facility (approximately 5 min), was associated with a lower probability of delivering at a facility in the order of at least 30 %. Five minutes travel time is roughly equivalent to 1 km distance at the travel speeds assumed appropriate to traffic conditions in Sylhet. This finding retained its significance even after allowing for intra-cluster correlation, since the use of clustered standard errors did not affect the significance levels of the estimated coefficients in either logistic or multinomial models. Remarkably, the decrease in utilization of facilities for delivery associated with increasing travel time in urban Sylhet was substantially higher than what has been observed in rural settings for distances that are far greater. In rural Ghana, for example, a one hour increase in travel time resulted in a 24 % decrease in facility utilization [24], and a 1 km increase in walking distance in rural Ethiopia resulted in a 22 % drop in the likelihood of delivering at a facility [28]. The reasons for the strong deterrent impact of travel time in urban areas may have a great deal to do with access. Amongst the urban poor population of Bangladesh, few possess reliable means of transportation suitable for getting women in labor to a facility. The Sylhet health seeking behavior survey cited earlier (Islam R et al., Health care seeking in poor urban settlements in Sylhet City Corporation- a quantitative survey. 2014. icddr,b & GIZ. Unpublished report), found that 86 % of women who had recently delivered at a facility got there by using natural gas-powered 3-wheelers or rickshaw for transport. Moreover, the poor urban households that constitute the focus of this study tend to be located in areas with poor road infrastructure and narrow alleys that are difficult to reach by emergency vehicles. Lastly, public emergency obstetric facilities providing free services that are commonly used by poor women are very few in number, and therefore, given the transportation challenges described above, even small differences in travel times can be perceived as insurmountable.

Consistent with the majority of studies examining the determinants of facility utilization for delivery in LMICs, women’s formal education and use of antenatal care were found to be other major determinants of whether a poor urban woman delivers at a facility or not (see systematic reviews [11, 19]). The impact of religious persuasion was less consistent, with mixed effects reported in the existing literature [11]. The results of this study most closely align with those in African settings where members of traditional religions and those of Muslim faith are less likely to use delivery services as compared to other religions persuasions [52, 53]. Higher age at first birth, stillbirth and involvement in paid employment also increased the likelihood of facility delivery, although to a lesser extent.

Analysis was extended by differentiating EmOC facilities into those that are privately, publicly and NGO managed. Interesting and previously unrecognized differences in the impact of geographic location, as well as socio-cultural and economic factors on the choice of delivery emerged from this study. Delivery at private facilities was more significantly affected by travel time, with a 5-min increase in travel time resulting in a 32.9 % decrease in the likelihood of private facility delivery, compared to a 28.2 and 28.6 % decrease in public and NGO facility deliveries for the same time interval. The stronger effect of geographic distance on delivery at private facilities was observed consistently throughout the analysis, most likely reflecting the deterrent effect of the preference for low-cost services among the urban poor recorded during the health-seeking behavior survey. Factors associated with increased likelihood of delivery at a private facility were mother’s access to media, previous stillbirth and lower number of living children. Moreover, results showed that women belonging to Muslim households were much less likely to deliver at any formal facility than non-Muslim mothers, and even more so at private facilities, with home delivery being the preference of most. Finally, use of antenatal care and mother’s education were also higher among women delivering at private facilities. Interestingly, choice of delivery at NGO facilities was found to be significantly related to unwanted pregnancies, while no such correlation was observed for public and private facilities. Although the impact of desire for pregnancy on the utilization of delivery services is less studied than education or antenatal care [11], our result evidenced a stronger impact than that found in existing literature [29, 54–56].

A limitation of this study was the availability of household geo-location at the aggregated cluster versus individual household level, which may have limited variation in our primary explanatory factor, ‘travel time to the nearest EmOC facility’. While this could be averted if point locations of individual households were available, given the small size of the clusters (a few 100 m at most) relatively to the distances between cluster centers and EmOC facilities (in the order of kilometers) it is unlikely that the use of sub-cluster travel time would significantly impact the results presented. Another limitation of this study is that average road speeds were used in the calculation of travel times, which does not necessarily reflect diurnal and seasonal variations in traffic conditions. This may have affected effective (or perceived) travel times to some extent. Lastly, in the absence of data on the actual facility used during delivery, an assumption was made that delivery would take place at the geographically closest facility of the type declared during interviews. This assumption may have been incorrect in certain cases and, in this context, might warrant further investigation.

## Conclusions

This study furnishes unique insights on how travel time to the nearest EmOC facility influences the choice of facility delivery among women living in urban poor settlements in Bangladesh. Findings support a number of intervention strategies to promote the use of EmOC facilities and reduce maternal and neonatal death due to life-threatening complications. Supported by geospatial evidence, one of these is to reduce travel time between poor population clusters and EmOC facilities facilities by establishing or relocating facilities closer to where the poor reside. Community planning and funding for emergency transport is another option that has been tried in rural northwestern Bangladesh, demonstrating promising success in terms of increasing EmOC utilization [57]. However, among the most effective actions to promote use of EmOC facility delivery across the health system (public, private and NGO) is increasing coverage of antenatal care and promoting female education. The fact that access to media (radio, TV and magazines) only positively impacts the likelihood of delivery at private facilities suggests a biased effect of information delivered through these mechanisms that works to enhance perceived quality of care in the private sector. It also emphasizes the need for active regulation to ensure that service quality in the private sector meets minimum standards of care.

Further studies should be carried out in other urban areas to assess whether similar patterns are apparent. Moreover, the same analysis could be applied to other types of services for which travel time is a critical factor (e.g., emergency services) to provide evidence of the impact of geographic location on healthcare utilization across the urban health system.

## Abbreviations

ANOVA: ANalysis Of VAriance
CI: Confidence interval
EmOC: Emergency obstetric care
LMIC: Low and middle income countries
MDG: Millennium development goal
MMR: Maternal mortality ratio
NGO: Non-government organizations
SCC: Sylhet City Corporation

## Footnotes

1 Variable ‘mother’s age’ is not included as covariate of the binomial logistic regression (Table 2) on grounds of posing a collinearity problem (based on collinearity diagnostics). However, this poses no loss of information since the parameter was found insignificant in the statistical analysis.

2 Due to collinearity problem affecting model convergence, variable ‘antenatal care usage’ could not be included in multinomial regression model (Table 4). All other covariates, however, were included.
