# Erratum to: The influence of travel time on emergency obstetric care seeking behavior in the urban poor of Bangladesh: a GIS study

**DOI:** 10.1186/s12884-016-1082-x

**Published:** 2016-09-27

**Authors:** Rocco Panciera, Akib Khan, Syed Jafar Raza Rizvi, Shakil Ahmed, Tanvir Ahmed, Rubana Islam, Alayne M. Adams

**Affiliations:** 1 International Centre for Diarrhoeal Disease Research, Bangladesh (icddr,b), 68 Shahid Tajuddin Ahmed Sharani, Mohakhali, Dhaka, 1212 Bangladesh; 2 James P. Grant School of Public Health, BRAC University, 5th Floor, (Level-6), icddr,b Building, 68 Shahid Tajuddin Ahmed Sharani, Mohakhali, Dhaka, 1212 Bangladesh; 3 Institute of Development Studies (IDS), University of Sussex, Library Road, University of Sussex, Brighton, East Sussex BN1 9RE UK

## Erratum

In the original publication of this article [1], one of the co-authors’ names contained a spelling error. ‘Alayne M. Adam’ should therefore have been listed as ‘Alayne M. Adams’. The original publication has now been modified as well to reflect these changes.
